# Chemotherapy-related complications in the kidneys and collecting system: an imaging perspective

**DOI:** 10.1007/s13244-015-0417-x

**Published:** 2015-07-11

**Authors:** Jemianne Bautista Jia, Chandana Lall, Temel Tirkes, Rajesh Gulati, Ramit Lamba, Scott C. Goodwin

**Affiliations:** Department of Radiology, University of California, Irvine School of Medicine, 101 The City Drive South, Mail Code: 5005, Orange, CA 92868 USA; Department of Radiology, Indiana University School of Medicine, 714 N. Senate Avenue, Suite 100, Indianapolis, IN 46202 USA; Department of Internal Medicine, University of California, Irvine School of Medicine, 101 The City Drive, Mail Code: 4076, Orange, CA 92868 USA; Department of Radiology, University of California, Davis School of Medicine, 2315 Stockton Blvd., Sacramento, CA 95817 USA

**Keywords:** Chemotherapy, Biologic agents, Nephrotoxicity, Renal cysts, Drug-associated adverse effects

## Abstract

**Abstract:**

Nephrotoxicity is a common adverse effect of many chemotherapeutic agents. The agents most commonly associated with chemotherapy-associated nephrotoxicity are methotrexate, semustine, streptozocin, mithramycin, and cisplatin. Certain chemotherapeutic agents have adverse effects on the kidneys and urothelium that can be visualized radiographically, including cystic change, interstitial nephritis, papillary necrosis, urothelial changes, haemorrhagic cystitis, acute tubular necrosis, and infarction. This review focuses on imaging features identifying complications of chemotherapy in the kidneys and collecting system and provides didactic cases to alert referring clinicians.

***Teaching Points*:**

• *Nephrotoxicity is a common adverse effect of many chemotherapeutic agents*.

• *Chemotherapies have adverse renal and urothelial effects that can be visualized radiographically*.

• *Crizotinib use can result in the development of complex renal cysts*.

## Introduction

Nephrotoxicity is a common adverse effect of many chemotherapeutic agents. A major contributing factor is the renal excretion of the majority of these agents. Damage is often identifiable clinically by changes in glomerular filtration rate, creatinine clearance, blood urea nitrogen, urine protein, and urine output. The most common agents causing chemotherapy-associated nephrotoxicity include cisplatin, methotrexate, mithramycin semustine, and streptozocin. Certain chemotherapeutic agents have adverse renal and urothelial effects that can be visualized radiographically including cystic change, interstitial nephritis, papillary necrosis, urothelial changes, haemorrhagic cystitis, acute tubular necrosis (ATN), and infarction. This review focuses on imaging features identifying complications of chemotherapy in the kidneys and collecting system and provides didactic cases to alert referring clinicians (Table 1).

**Table 1:** Table summarizing adverse effects in the kidneys and collecting system visible on imaging and associated cancer therapies

Adverse effect	Associated agents	Laboratory findings	Radiologic findings
Complex renal cysts	Crizotinib	None	*US*- Ovoid, anechoic cysts with clearly demarcated walls, no septa or calcifications, near water density
			*CT*- Bosniak III-IV complex cysts
Interstitial nephritis	Ipilimumab	↑Plasma creatinine	*Urography*- Enlarged kidneys, dense persistent nephrogram
	Sorafenib	Azotemia	
		FENa> 1 %	*US*- Enlarged kidneys
		Eosinophiluria	
		Proteinuria	*CT*- Renal edema and enlargement, streaky parenchymal low-attenuation areas
		Pyuria	
Renal papillary necrosis	Nedaplatin	↑ Plasma creatinine	*Urography*- Irregular contour of the renal papillae and widening of the fornixes, “ball on a tee sign”
		Azotemia	
		Leukocytosis	
		Hematuria	
		Proteinuria	*US*- Multiple cystic spaces in the medullary region arranged around the renal sinus, non-shadowing soft tissue masses within the ureter
		Pyuria	
			*CT*- Excavation of the calyces, regression of the papillae, blunting of the calyces, detached papillae in the ureter
Renal infarction	Methotrexate	↑ Lactate dehydrogenase Hematuria	*Urography*- Absence of contrast material in infarcted parenchyma
	Combination Cisplatin and Gemcitabine Regimens		
		Proteinuria	
			*US*- Heterogeneity of parenchyma
			*CT*- Low attenuation, wedge shaped areas in the cortex, “rim sign”
Acute tubular necrosis	Cisplatin	↓ GFR	*Urography*- Renal enlargement with prolonged opacification of the renal parenchyma, increase in density of the pyramids
	Ifosfamide		
	Imatinib		
			*US*- Cortical echogenicity
			*CT*- Contrast retention in the parenchyma, “rim sign”
		↓ Urine osmolality	
		↓ Urine/plasma creatinine ratio	
		Urine sediment: renal tubular epithelial cells, epithelial cell casts, and muddy brown granular casts	
Chemotherapy cystitis	Intravesical Mitomycin C	Hematuria	*Urography*- Small bladder with thickened walls, calcifications within the walls may be present
			*CT*- Diffuse or focal irregular bladder wall thickening, decreased bladder volume and perivesical fat, edema
			*MRI*- High T2 signal within the bladder wall
Hemorrhagic cystitis	Cyclophosphamide	Hematuria	*CT*- Bladder wall thickening
	Ifosphamide		
	Busulfan		
	Cabazitaxel		

## Effects of chemotherapeutic agents on cross-sectional imaging of the kidney

### Complex renal cysts

The development of complex renal cysts associated with crizotinib treatment is well documented in the literature. Crizotinib is an anaplastic lymphoma kinase (ALK) inhibitor used in the treatment of ALK positive metastatic non-small cell lung cancer (NSCLC). Lin et al. explored the presence of complex renal cysts in patients enrolled in prospective clinical trials for crizotinib treatment. In the 32 patients included in the study, 13 % developed new complex renal cysts and 22 % had significant renal cyst change following initiation of crizotinib treatment. After cessation of crizotinib treatment, the cysts were found to regress significantly [1]. In a similar retrospective study, Schnell et al. reported that of 17 patients found with complex renal cysts associated with crizotinib use, 11 required hospitalization due to the cysts, with seven having cystic invasion into adjacent structures in the form of inflammatory cystic masses. The majority of patients were asymptomatic, but a small number presented with flank pain or fevers [2].

#### Imaging findings

Urography cannot be used to definitively diagnose complex cysts as they present as indiscernible masses when this technique is used [3]. Sonographically, cysts associated with crizotinib have been described as ovoid, anechoic with internal echoes, near-water density, with smooth clearly demarcated walls and acoustic enhancement behind the cysts, and without septa or calcifications [4]. On ultrasound (US), diagnosis of cysts may be complicated by a number of factors. Vascular malformations or aneurysms could be mistaken for cystic disease if real-time studies do not demonstrate pulsations or large feeding vessels are not delineated. In addition, peripelvic cysts often contain artificially created echoes due to their proximity to structures of the collecting system and need to be confirmed on computed tomography (CT) [3].

**Fig. 1:**
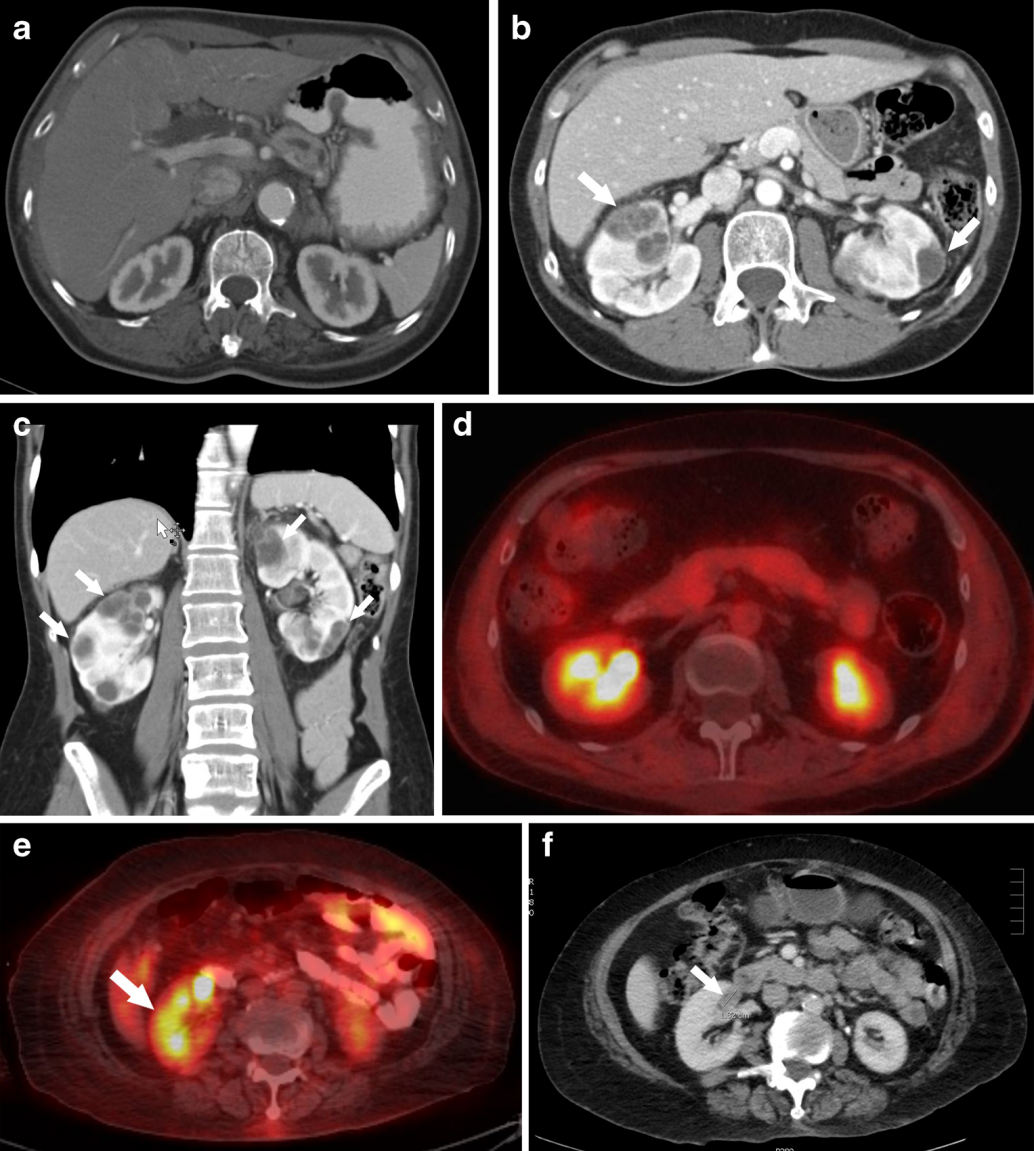
Forty-nine-year-old female with NSCLC being treated with long-term crizotinib therapy. (**a**) Pre-treatment axial CT with normal renal findings. (**b**, **c**) Axial and coronal CT images, respectively, performed 3 years following initiation of treatment with crizotinib revealing multiple complex cystic lesions bilaterally (*arrows*). (**d**) Axial positron emission tomography (PET)/CT without evidence of abnormal FDG-avid uptake supporting a benign process. (**e**, **f**) Axial PET/CT of a different patient with abnormal FDG-avid uptake positive for renal neoplasm provided as comparison (*arrows*)

On CT, complex renal cysts can be distinguished from benign cysts using criterion laid out by Bosniak. The presence of extensive calcification, septa with irregular walls thicker than 1 mm or associated with solid elements at their attachments, hyperdense fluid with irregularity of contour or hazy margination, internal haemorrhage or debris, and thickening or irregularity of the wall or any evidence of solid tissue within the wall distinguish complex from benign cysts (Fig. 1a–c) [3]. Cysts associated with crizotinib use have been reported as Bosniak classification types II-IV [1, 4]. Bosniak II cysts are well marginated and characterized by a few thin septa less than 1 mm and thin calcifications. Bosniak III cysts are characterized by thick or multiple septations, mural nodule, and are hyperdense on CT. Bosniak IV cysts are characterized as solid masses with large cystic or necrotic components, irregular margins, and solid vascular elements [2]. Complex cysts may mimic metastatic disease and it is thus important to have knowledge of this entity (Fig. 1d–e). On examination of cysts with CT, it is important to obtain pre- and post-contrast imaging so that calcifications and recent haemorrhage can be identified and so high-density non-enhancing renal cysts are not mistaken for solid metastatic lesions. If enhancement is present, renal abscesses and metastases are included in the differential. It is imperative for diagnosis on CT that the fluid in the cyst is near water density with a suggested upper threshold of 20 HU. If the density exceeds 20 HU, other pathologies such as tumours need to be considered [3]. CT and magnetic resonance imaging (MRI) findings of complex renal cysts are equivalent with the two techniques resulting in similar ratings based on the Bosniak system [5].

### Interstitial nephritis

Interstitial nephritis refers to inflammation of the renal interstitium. Histologically, this is characterized by interstitial infiltration by lymphocytes, monocytes, and granulomas. Symptoms may include oliguria and less commonly, hematuria. Patients may also have nonspecific symptoms of fever, rash, and loin pain, but in many cases patients are asymptomatic [6]. Interstitial nephritis can be associated with a decline in creatinine clearance, eosinophilia, eosinophiluria, and proteinuria. Multiple chemotherapy regimens have been associated with interstitial nephritis, most notably ipilimumab. In ipilimumab-induced interstitial nephritis, patients are treated with prednisone and quickly return to their baseline kidney function [7].

#### Imaging findings

**Fig. 2:**
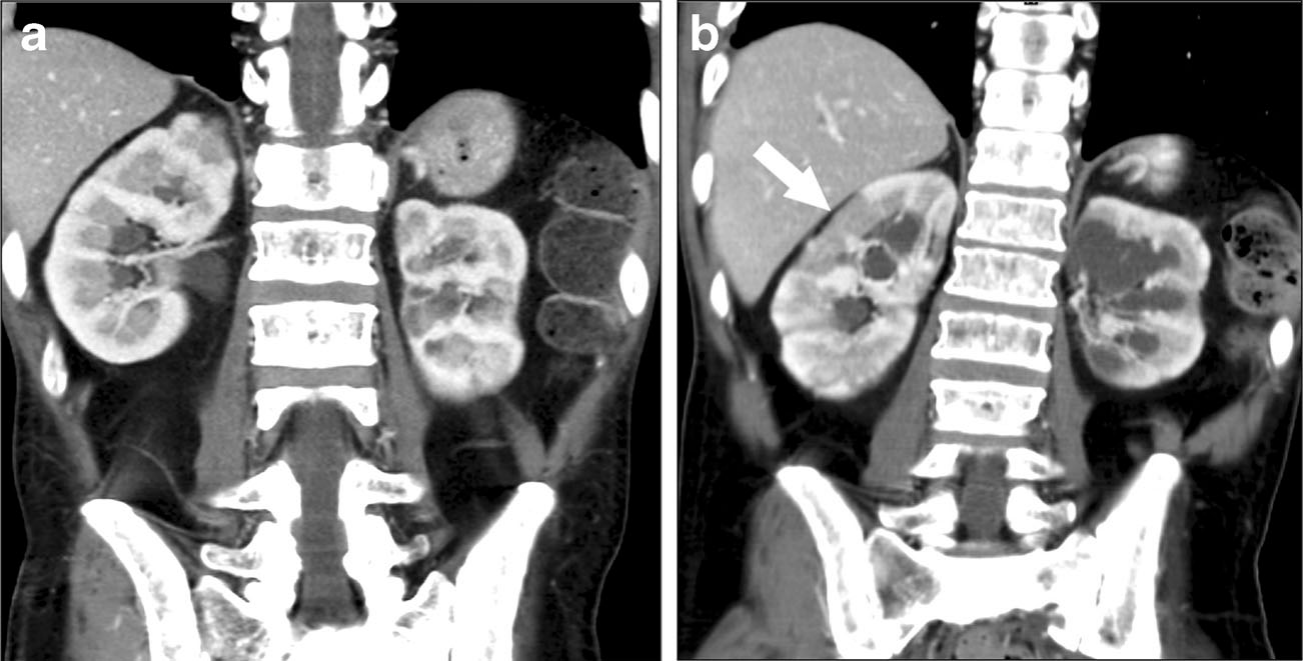
Forty-three-year-old male with soft tissue sarcoma, on a combination high-dose cisplatin chemotherapy regimen. (**a**) Pre-chemotherapy coronal CT showing normal renal findings. (**b**) Post-chemotherapy coronal CT showing an interstitial nephritis pattern with enlargement of the kidneys and multiple hypo-attenuating lesions, more prominent in the right kidney (*arrow*)

**Fig. 3:**
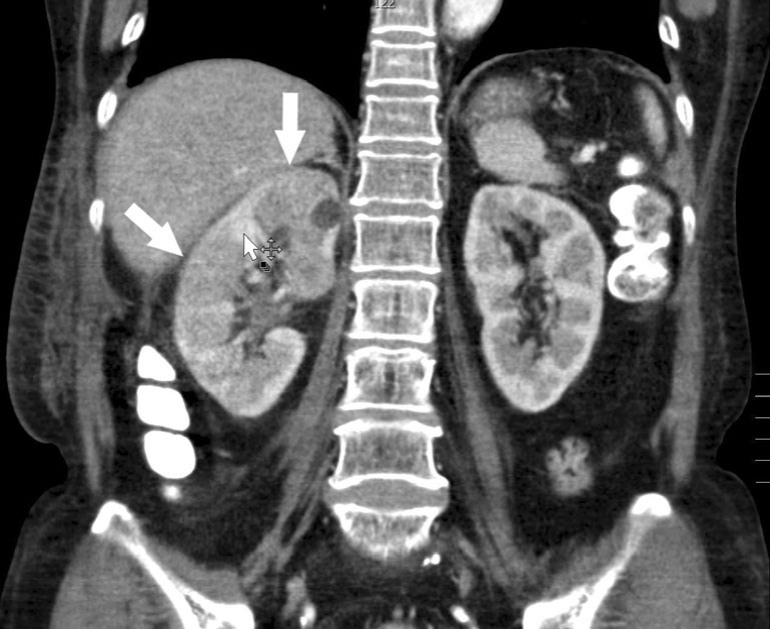
Sixty-five-year-old female with multiple myeloma being treated with a combination chemotherapy regimen that included carmustine. Post-chemotherapy CT showing bilateral enlarged kidneys and a hypo-attenuating lesion in the right kidney (*arrows*) consistent with interstitial nephritis

On urography, acute interstitial nephritis often reveals enlarged kidneys as well as an early, dense, persistent nephrogram, which is similar to the findings of ATN. An important distinguishing characteristic is that ATN results in normal sized or only slightly enlarged kidneys [8]. On US imaging, acute interstitial nephritis may not be apparent or may present with enlargement of the kidneys as well [9]. CT findings include renal oedema and enlargement. The presence of streaky parenchymal low-attenuation areas, which may mimic more common entities like pyelonephritis and multifocal infarcts among others, may also be seen (Figs. 2a, b, and 3) [10]. Radiographic findings are not diagnostic of interstitial nephritis and findings need to be confirmed with biopsy. However, imaging is useful in excluding obstruction that would require decompression.

### Renal papillary necrosis

Renal papillae are present at the apex of the renal pyramid at the site where urine is discharged from the renal tubules. Papillary necrosis occurs secondary to ischemia and can be triggered by a number of factors, the most common ones being analgesic nephropathy, diabetes, sickle cell disease, and infection. Histologically, renal papillary necrosis (RPN) appears as coagulative necrosis, characterized by a pale centre with surrounding inflammatory cells [11]. Symptoms of RPN most commonly include fevers, chills, flank pain, and hematuria. Sloughed papillae can cause ureteral obstruction and hydronephrosis, further worsening renal function. The platinum based agents, cisplatin and nedaplatin, are associated with RPN [12]. Care needs to be taken in interpretation of this pathological outcome as it can be difficult to determine whether the cause is the chemotherapy itself or chronic analgesic use since cancer patients are often on powerful analgesic regimens.

#### Imaging findings

**Fig. 4:**
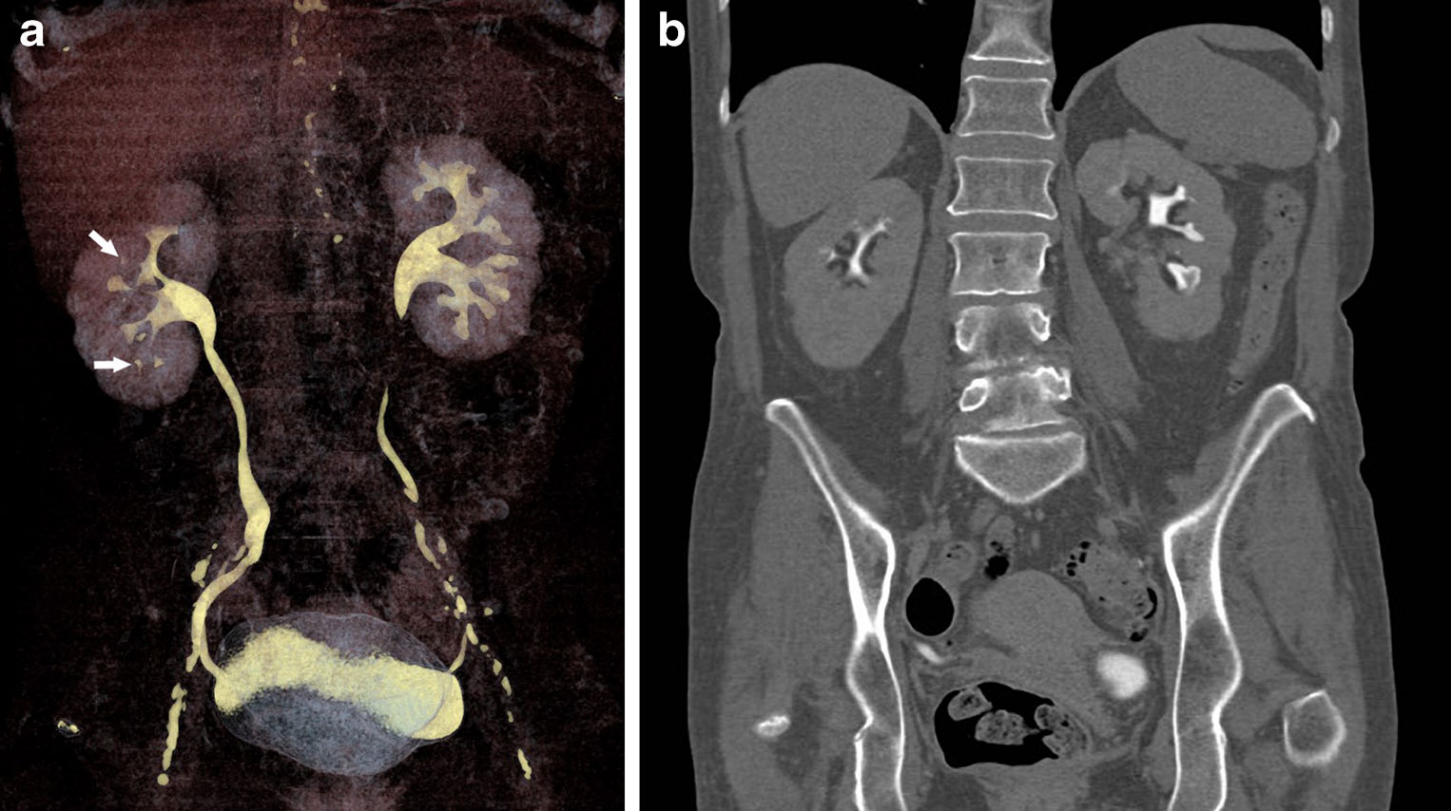
Sixty-two-year-old woman with node positive ductal carcinoma undergoing treatment with carboplatin and paclitaxel. (**a**) Coronal maximum intensity projection (MIP) image showing bilateral blunting of the calices consistent with renal papillary necrosis. (**b**) CT-urographic image demonstrating bilateral irregularly shaped, projections at the apex of the left renal pyramids suggestive of papillary necrosis

Urographic findings are diagnostic with irregular contour of the renal papillae and widening of the fornixes (Fig. 4a). After sloughing of the papilla, contrast penetrates the renal parenchyma and ring shaped shadows appear to demonstrate the detached papilla, the so-called ball on a tee sign [13]. Sonographically, renal papillary necrosis appears as multiple cystic spaces in the medullary region arranged around the renal sinus echoes [14]. Necrosed papilla appear as nonshadowing soft tissue masses within the ureter which cannot be differentiated from other pathologies such as blood clots [14]. Hydronephrosis which has similar findings can be differentiated from renal papillary necrosis by the presence of a central dilation of the pelvis [15]. On CT, renal papillary necrosis is characterized by excavation of the calyces, regression of the papillae, detached papilla in the ureter, and blunting of the calyces (Fig. 4b) [16].

### Renal infarction

Renal infarction presents with persistent pain often resistant to analgesia, nausea and vomiting, proteinuria, hematuria, and elevated lactate dehydrogenase [17]. In the literature, it has been reported with use of methotrexate and combination regimens of cisplatin and gemcitabine [18, 19]. In a case series with 44 patients with long-term follow-up for renal infarction, 61 % of patients regained normal renal function while the remaining patients alive at follow-up had progressed to irreversible kidney dysfunction [20].

#### Imaging findings

**Fig. 5:**
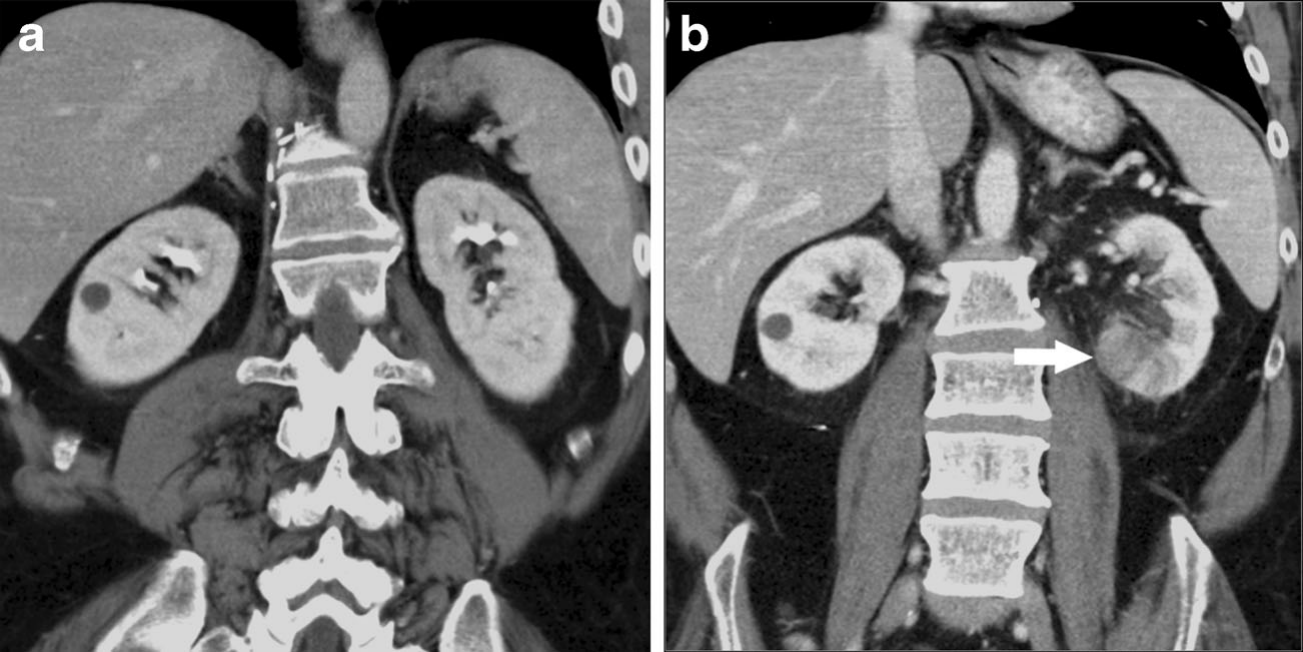
Fifty-four-year-old male with testicular cancer presenting with evidence of renal infarct following four cycles of bleomycin, etoposide, and cisplatin. (**a**) Pre-treatment coronal CT showing normal appearance of the kidneys. (**b**) Post-treatment coronal CT showing developing left renal infarct appearing as an area of hypoattenuation in the inferior aspect (*arrow*)

Urography findings for renal infarction vary according to the severity. Small infarcts often present with normal urograms. With more severe lesions, there can be an absence of contrast material in the ischemic renal parenchyma with local failure of calyceal filling of the affected tissue during the pyelographic phase. Renal infarction may also result in vasospasm which presents as complete nonvisualization of the kidney [21]. On US, acute renal infarcts may be non-specific in appearance, with heterogeneity of the parenchyma. Chronic infarcts may be wedge shaped and hypoechoic with cortical scarring [22]. On CT, renal infarcts appear as low attenuation, wedge shaped areas in the cortex (Fig. 5a, b). Sometimes the “rim sign” is present, which is a higher-attenuation subcapsular rim surrounding lower-attenuation infarcted renal parenchyma representing subcapsular perfusion through collateral flow [23, 24]. However, as the “rim sign” is a universal and highly specific indicator of renovascular compromise, it can also be present in acute tubular necrosis and renal vein thrombosis. Hydronephrosis similarly is characterized by a “rim sign”; however, this rim sign can be distinguished from that of vascular compromise by its variable thickness, medial concavity, and location surrounding the dilated calices in communication with the central pelvis [25].

### Acute tubular necrosis

ATN refers to acute renal failure caused by an ischemic or toxic insult to the tubular epithelial cells. This results in epithelial cell detachment from the basement membrane causing tubular dysfunction. It presents with a decrease in GFR, urine osmolality, and urine/plasma creatinine ratio. Urine sediment is characterized by renal tubular epithelial cells, epithelial cell casts, and muddy brown granular casts [26]. ATN is associated with cisplatin and ifosfamide treatment [27, 28]. Patients may return to baseline kidney function following ATN; however, some patients develop an irreversible decline in function. Risk factors for permanent injury include age over 65 years, atheroembolic disease, and preexisting chronic kidney disease. ATN that develops in the hospital setting is associated with a high mortality rate [29, 30].

#### Imaging findings

**Fig. 6:**
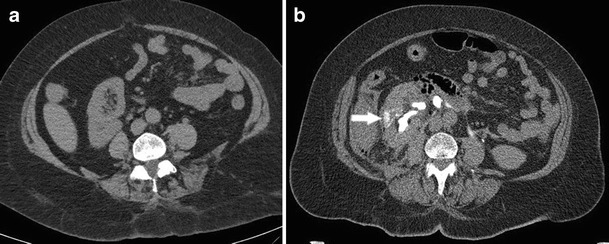
Eighty-year-old woman with gastric cancer mid-treatment with cisplatin and 5-FU. (**a**) Pretreatment axial CT demonstrating normal renal characteristics. (**b**) Noncontrast axial CT demonstrating contrast retention in the right renal parenchyma consistent with ATN (*arrow*)

On urography, acute tubular necrosis appears as renal enlargement with prolonged opacification of the renal parenchyma and an increase in density of the pyramids [31]. On sonography, ATN presents as increased cortical echogenicity [32]. Inadequacy of perfusion can also be detected as abnormal Doppler velocity waveforms [33]. On CT imaging, ATN presents with contrast retention in the parenchyma (Fig. 6a, b). The “rim sign” can also be used to characterize ATN and is a valuable factor distinguishing infarction from pyelonephritis [25].

## ‬‬‬‬‬Effects of chemotherapeutic agents on cross-sectional imaging of the collecting systems‬‬‬‬‬‬‬‬‬‬‬‬‬‬‬‬‬‬‬‬‬‬‬‬

### Chemotherapy-induced cystitis

Chemotherapy-induced cystitis is characterized by epithelial proliferation following chemotherapy treatment. A study looking at 17 cases of both chemotherapy- and radiation-induced cystitis histologically characterized the cystitis as epithelial proliferation within the lamina propria with mild to moderate nuclear pleomorphism without mitoses. Pseudoinvasive urothelial nests wrapping around vessels, haemorrhage, fibrin, deposition, and acute and chronic inflammation were also present in all cases. All patients presented with hematuria with the presentation of symptoms occurring anywhere from mid-chemotherapy treatment to as far as 60 days following treatment cessation in patients with chemotherapy-induced cystitis. All patients available for follow-up at 9 months had an improvement in their hematuria and 71 % had negative cystoscopies at this time. No patients went on to develop bladder cancer [34]. Chemotherapy cystitis is associated with intravesical use of mitomycin C [35].

#### Imaging findings

Radiologic findings of chemotherapy-induced cystitis are nonspecific and cannot be distinguished from other causes of cystitis. On intravenous urography and cystography, the bladder may be small with thickened walls. Rarely, calcifications can be present within the wall [36]. On CT imaging, acute chemotherapy-induced cystitis may present with diffuse or focal irregular bladder wall thickening, decreased bladder volume and perivesical fat, and oedema. Increased contrast enhancement of the bladder wall is not usually present [36]. On MRI, there is high T2 signal intensity within the bladder wall, which is suggestive of inflammation [37]. Increased signal intensity of the mucosa may also be seen on T1-weighted images and is likely attributable to mucosal haemorrhage [36].

### Hemorrhagic cystitis

Hemorrhagic cystitis is inflammation of the bladder that is characterized by mucosal hyperemia, ulcerations, haemorrhage, and necrosis. Symptoms consist of hematuria, frequency, dysuria, burning, urgency, incontinence, and nocturia [38]. Hemorrhagic cystitis is highly associated with oxazaphosphorine compounds, especially cyclophosphamide and ifosphamide [39]. Busulfan [40] and cabazitaxel [41] are also implicated. Mesna and continuous bladder irrigation and hyperhydration have been shown to be effective in preventing cyclophosphamide-induced hemorrhagic cystitis [42, 43]. Hemorrhagic cystitis caused by chemotherapeutic agents is generally reversible following cessation of the offending agent.

#### Imaging findings

**Fig. 7:**
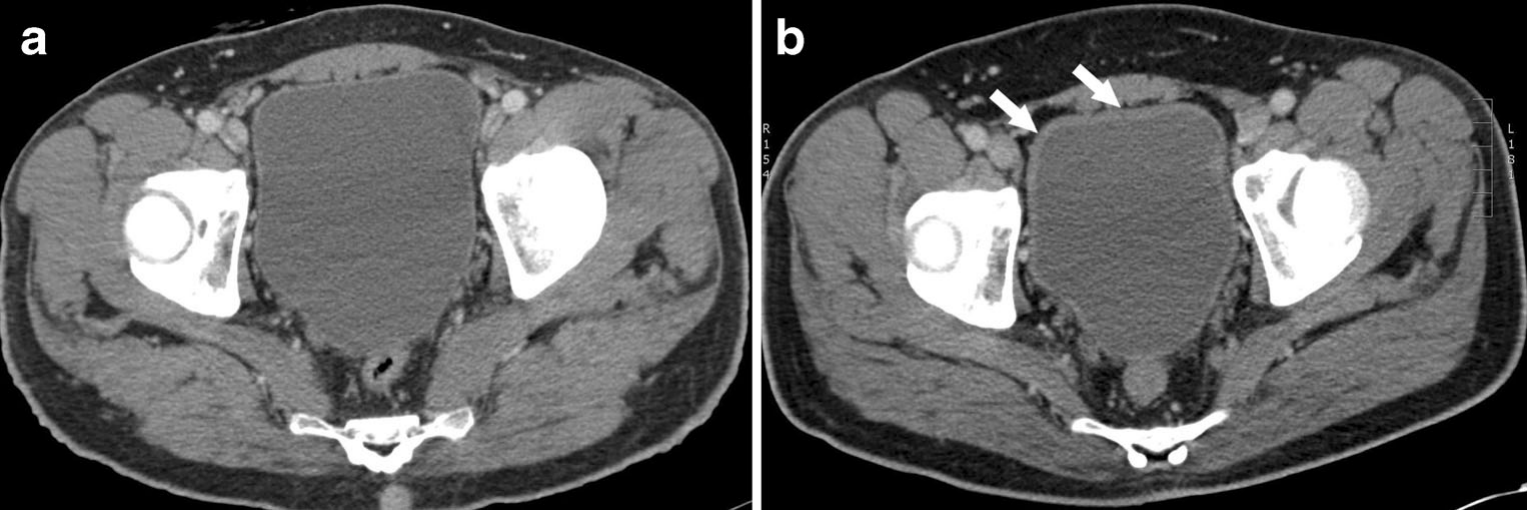
Forty-four-year-old male with non-Hodgkin’s lymphoma status post treatment with cyclophosphamide. (**a**) Axial CT pre-chemotherapy image showing normal bladder findings. (**b**) Axial CT image showing thickened urinary bladder wall consistent with chemotherapy induced hemorrhagic cystitis (*arrows*)

Excretory urograms can be normal in hemorrhagic cystitis [38]. US is a useful technique in evaluation of hemorrhagic cystitis as power Doppler allows estimations of the hypervascularity, and thus the severity of the condition [44]. Hemorrhagic cystitis appears as bladder wall thickening on CT (Fig. 7a, b) [45]. However, as the imaging characteristics are not specific, they need to be correlated clinically with hematuria, which is the most common presenting symptom.

## Effects of novel targeted agents on the kidney and collecting systems

Cancer therapy has been moving in the direction of targeted therapies. As evidence surfaces for these novel agents, many are found to have renal toxicities. Antiangiogenic compounds such as bevacimumab, sunitinib, and sorafenib, which inhibit the VEGF pathway, are associated with proteinuria, increased levels of creatinine, and hypertension. MET inhibitors in phase II trials have also been found to be associated with proteinuria and hypertension. The EGFR inhibitors, erlotinib, cituximab, and gefitinib, and the HER2 inhibitors, trastuzumab and lapatinib, have not been associated with any renal toxicities.

Adverse effects associated with newer targeted agents visible radiographically include interstitial nephritis reported with sorafenib treatment and tubular necrosis reported with imatinib treatment [46].

## ‬‬‬‬‬‬‬‬‬‬‬‬‬‬‬‬‬‬‬‬‬‬‬‬Conclusion

Chemotherapy is vital in the treatment of cancer. However, its inherent toxicity often has unintended adverse effects. The kidney and collecting system are commonly involved due to their role in filtration and elimination. Chemotherapy can cause various changes including complex renal cysts, interstitial nephritis, urothelial irritation, acute and chronic tubular necrosis, and renal infarction, among others. Aside from complex renal cysts associated with crizotinib use, the pathologies elaborated on in this manuscript are often symptomatic and can negatively impact renal or collecting system function. Therefore, if imaging suggestive of these pathologies arises, renal function tests need to be performed and regimens adjusted accordingly. With complex cysts secondary to crizotinib use, regression occurs spontaneously following treatment. However, if patients are symptomatic or if invasion into surrounding structures is noted on imaging, then further evaluation and medication adjustments need to be made. Familiarization with imaging features consistent with post-chemotherapy changes to the kidneys and urothelium and subsequent recognition and reporting will improve clinical monitoring of patients, early detection of chemotherapy-associated complications, and will ultimately result in improved patient care and outcomes through modification, interruption, or suspension of therapy.
